# Cell-Free Culture Supernatant of *Lactobacillus acidophilus* AG01 and *Bifidobacterium animalis* subsp. *lactis* AG02 Reduces the Pathogenicity of NetB-Positive *Clostridium perfringens* in a Chicken Intestinal Epithelial Cell Line

**DOI:** 10.3390/microorganisms12040839

**Published:** 2024-04-22

**Authors:** Darshana Kadekar, Andreea Cornelia Udrea, Steffen Yde Bak, Niels Christensen, Kirsty Gibbs, Chong Shen, Marion Bernardeau

**Affiliations:** 1 Gut Immunology Lab, R&D, Health & Biosciences, IFF, 8220 Brabrand, Denmarkandreea.cornelia.udrea@iff.com (A.C.U.); 2 IFF Advanced Analysis, R&D, ET, IFF, 8220 Brabrand, Denmark; steffen.yde.bak@iff.com (S.Y.B.); niels.christensen@iff.com (N.C.); 3 Danisco Animal Nutrition, IFF, 2342 BH Oegstgeest, The Netherlands; kirsty.gibbs@iff.com; 4 Agro-Food Department, Normandy University, UNICAEN, ABTE, 14000 Caen, France

**Keywords:** probiotic, broilers, chicken intestinal epithelial cells, *Clostridium perfringens*, necrotic enteritis, NetB

## Abstract

The worldwide reduction in the use of antibiotics in animal feed is fueling the need for alternatives for the prevention and control of poultry intestinal diseases such as necrotic enteritis (NE), which is caused by *Clostridium perfringens*. This is the first report on the use of an intestinal epithelial chicken cell line (CHIC-8E11) to study the pathogenic traits of *C. perfringens* and to investigate the mode of action of cell-free supernatants (CFS) from probiotic *Lactobacillus acidophilus* AG01 and *Bifidobacterium animalis* subsp. *lactis* AG02 in reducing the pathogenicity of *C. perfringens*. The cell adhesion, permeability and cytotoxicity were assessed under challenge with four *C. perfringens* strains isolated from broiler NE episodes of differing geographical origin (CP1–UK; CP10–Sweden; 25037–CP01 and CP22–USA). All the *C. perfringens* strains could adhere to the CHIC-8E11 cells, with varying affinity (0.05–0.48% adhesion across the strains). The CFS from one out of two strains (CP22) increased the cell permeability (+4.5-fold vs. the control, *p* < 0.01), as measured by the fluorescein isothiocyanate-dextran (FD4) content, with NetB toxin implicated in this effect. The CFS from all the strains was cytotoxic against the CHIC-8E11 cells in a dose- and strain-dependent manner (cytotoxicity 23–62% across the strains when dosed at 50 µL/mL, as assessed by the MTT cell viability assay). Pre-treatment of the cells with CFS from *B. animalis* subsp. *lactis* AG02 but not *L. acidophilus* AG01 reduced the cell adhesion of three out of four *C. perfringens* strains (by 77–85% vs. the control, *p* < 0.001) and reduced the negative effect of two NetB-positive strains on the cell permeability. The CFS of both probiotics alleviated the cytotoxicity of all the *C. perfringens* strains, which was dependent on the dose. The results confirm the suitability of the CHIC-8E11 cell line for the study of host–pathogen cell interactions in the context of NE caused by *C. perfringens* and reveal a beneficial mode of action of *B. animalis* subsp. *lactis* AG02 in reducing *C. perfringens* cell adhesion and, together with *L. acidophilus* AG01, in reducing *C. perfringens* cytotoxicity.

## 1. Introduction

*Clostridium perfringens* (*C. perfringens*) is a Gram-positive anaerobic bacterium that is a commensal member of the intestinal microbiota of poultry but is also the causative agent of necrotic enteritis (NE). In the presence of certain pre-disposing factors, *C. perfringens* can over proliferate and become pathogenic. For example, when there is an imbalance in the composition of the intestinal microbiota, such as may be caused by changes in diet composition [1], the use of therapeutics [2], or other host and environmental factors [3]. Necrotic enteritis is a major disease in poultry. It affects ~40% of commercial broiler flocks, causing diarrhea and high mortality in 2–5-week-old birds and costing the global broiler industry up to USD six billion annually [4]. *C. perfringens* strains can produce 18 or more different toxins [5], including α, β, ε and NetB toxins. These are secreted by the bacterium during disease pathogenesis. In vitro, they can be collected from bacterial cultures as part of the cell-free supernatant (CFS). The NetB toxin, produced by type G strains [6], has been identified as the key virulence factor for NE and can cause symptoms even in the absence of the pathogen itself [7]. No effective vaccines against NE are currently available. 

In the past, antibiotics and anticoccidials added to feed were used as prophylactic measures to control and prevent NE caused by *C. perfringens*. The recent prohibition of antibiotics in feed in major regions of the world and concern over *C. perfringens* emerging as a reservoir of antibiotic resistance genes [8] have fueled interest in the development of effective alternatives. The approaches studied to date variously rely on the positive modulation of the host immune response, improved intestinal barrier integrity, production of antimicrobials, pathogen inhibition and competitive exclusion. They include probiotics, prebiotics, postbiotics, short-chain fatty acids, plant extracts and essential oils [9,10,11,12,13,14]. 

Recently reclassified into 23 genera by Zheng et al. [15], *Lactobacillus* are among the major bacterial genera naturally present in the intestinal microbiota of poultry [16]. They have been identified as probiotic candidates because of the well-established beneficial effects on gut health and immunity as well as antimicrobial activity [17]. In broilers challenged orally with *C. perfringens*, it has been shown that *L. acidophilus* supplementation improves weight gain and reduces intestinal lesion scores, mRNA expression of pro-inflammatory cytokines, ileal populations of *Escherichia coli* and serum endotoxin content [18]. Further studies have shown that some *Lactobacillus* sp. produce antimicrobial compounds, including organic acids, antifungal peptides and bacteriocins, which alter the pathogen gene expression or otherwise inhibit pathogenic bacteria [19,20]. A range of probiotic effects of *Bifidobacterium* sp. in poultry have also been reported in numerous in vitro and in vivo studies [21,22], including in broilers challenged with *C. perfringens* or with *Salmonella typhimurium* [23,24]. The reported effects range from improved weight gain and other growth indicators, to pathogen exclusion, to increased production of inflammatory cytokines and certain blood serum parameters. These data have suggested a potential role for *Lactobacillus* and *Bifidobacterium* probiotics in reducing the pathogenicity of major poultry pathogens [25]. This has recently been tested in vivo in broilers in the context of a mild NE infection initiated by oral challenge with a NetB+ strain of *C. perfringens* [26]. The authors observed improved broiler performance (body weight and feed conversion ratio) and reduced NE lesions in birds supplemented with *L. acidophilus* AG01 and *B. animalis* subsp. *lactis* AG02 administered via the waterline, as compared to the challenged control, by 42 d of age.

The present study sought to investigate the in vitro mode of action of the two probiotic strains recently shown by van der Klein et al. [26] to have a beneficial effect on NE-challenged broilers in vivo. A novel chicken enterocyte cell line, CHIC clone 8E11 (Tentamus Pharma & Med Deutschland GmbH), was used in preference over established human (e.g., Caco-2) or pig (e.g., IPEC-J1 and IPEC-J2) cell models. This model is likely to be more representative of the poultry gut epithelial response to pathogen challenge due to the subtle but distinct differences between humans and poultry in the biochemical structure and functioning of the intestinal epithelial cell layer [27,28]. The CHIC-8E11 model has started to be applied to the study of pathogen–host cell interactions [29,30,31,32]; for example, to identify novel genes and mutations involved in host cell adhesion in pathogenic *E. coli* [30] and in *Salmonella enterica* isolates [31]. However, to date, only one study has used the model to study *C. perfringens*–host cell interactions, specifically to look at the production of the anti-inflammatory cytokine IL-10 by *C. perfringens*-challenged cells [33]. In the present study, we used the CHIC-8E11 cell line to evaluate the wider pathogenic effects of *C. perfringens* and the potential of probiotic bacteria to reduce those effects. We first confirmed the chicken origin and characteristics of the cell line and then used it to characterize the adhesion to host cells, pathogen exclusion, effects on cell permeability and the cytotoxicity of four *C. perfringens* strains and of pure NetB toxin. We then investigated the potential of CFS obtained from the probiotic strains *L. acidophilus* AG01 and *B. animalis* subsp. *lactis* AG02 to beneficially alter these effects.

## 2. Materials and Methods

### 2.1. Reagents and Materials

The NetB toxin was purchased from Prof. Filip Van Immerseel (Ghent University, Merelbeke, Belgium) at a concentration of 1 mg/mL. All the culture media, equipment and reagents were purchased from Thermo Fisher Scientific (Roskilde, Denmark), unless otherwise stated. 

### 2.2. Cell Line, Bacterial Strains and Culture Conditions

The CHIC-8E11 cell line was kindly donated by Prof. Dr. Peter Schierack and Dr. Muhammad Moman Khan of the Institute of Biotechnology, Brandenburg University of Technology, Senftenberg, Germany. The cells were grown in Dulbecco’s modified Eagle’s medium (DMEM)/Ham’s F-12 mixture (Sigma Aldrich, Dorset, UK) supplemented with 1 mM L-glutamine and penicillin–streptomycin and 10% fetal bovine serum, incubated at 37 °C in an atmosphere of 5% CO_2_ and passaged using standard protocols. 

Prior to its use in the experimental work, the CHIC-8E11 cell line was confirmed as being of chicken origin by using RT-PCR to evaluate the protein expression using chicken-specific PCR primers. For this preliminary work, the CHIC 8E11, IPEC-J2 (DSMZ, Braunschweig, Germany) and Caco-2 (DSMZ, Braunschweig, Germany) cells were cultured in DMEM, DMEM/F-12 and RPMI-1640, respectively, until confluency. The cells were lysed, RNA extracted, and RT-PCR performed in accordance with the procedures described by Shen et al. [34]. The results of this preliminary work are presented in Table S1 in the Supplementary Materials. The CHIC-8E11 cells expressed the major chicken epithelial cell proteins CDX1, CDH3, SOX9 and cytokine IL-8, which were not expressed in the human Caco-2 or porcine IPEC-J2 cells. In addition, the expression of proteins related to mesenchymal cells (vimentin and desmin), fibroblasts (ACTA-2) and stem cells (Lgr-5, Pcna and HopX) further confirmed the CHIC-8E11 model as being representative of the structural complexity of the poultry intestinal epithelium [35]. 

The *L. acidophilus* AG01 and *B. animalis* subsp. *lactis* AG02 were obtained from the Danisco Global Culture Collection (DGCC; Niebüll, Germany) and cultured on MRS broth (Man–Rogosa–Sharpe, BD Difco, Grenoble, France) at 37 °C in anaerobic conditions (Anaerocult, Merck, Darmstadt, Germany). 

Four *C. perfringens* strains, CP1, CP10, 25037-CP01 and CP22, originally isolated from infectious NE episodes, were obtained from the Danisco Culture Collection. All had previously been characterized as expressing at least one of two known virulence factors (a toxin and/or NetB toxin) based on whole-genome sequencing, as summarized in Table 1. All the strains were cultured in tryptic soy broth (TSB) at 37 °C in anaerobic conditions. 

### 2.3. Preparation of Cell-Free Supernatant (CFS)

The cell-free supernatant (CFS) from the *C. perfringens* strains was collected from overnight liquid cultures grown in brain heart infusion (BHI) broth at 37 °C under conditions of 5% CO_2_. This produced a suspension with a maximum optical density (OD) of 1.5 ± 0.3, as determined spectrophotometrically at 600 nm following calibration against a blank sample of the culture medium. The cultures were centrifuged at 4000× *g* for 20 min, and then the supernatants were removed and filtered through a 0.2 µm Nalgene filter to obtain the CFS. The CFS was stored at −20 °C until later use. 

The cell-free supernatant from *L. acidophilus* AG01 and *B. animalis* subsp. *lactis* AG02 was prepared from cultures grown in MRS for 48 h at 37 °C under conditions of 5% CO_2_. The absorbance of the resulting suspensions was read at 600 nm and the concentration adjusted to an OD of 1 by the addition of culture medium, as necessary. The bacterial suspensions were then plated on agar, grown and the colonies counted. It was determined that an OD of 1 was equivalent to a bacterial concentration of 1 × 10^7^ colony-forming units (CFU)/mL. This final concentration was used to prepare the CFS for each probiotic strain as described for *C. perfringens* above. These were stored under the same conditions as for the *C. perfringens* CFS.

### 2.4. Cell Adhesion Assay

Without probiotic pre-treatment: The cell adhesion assay was performed according to the protocol described by Letourneau et al. [36]. Briefly, isolated CHIC-8E11 cells were seeded in 96-well cell culture plates at a density of 2.0 × 10^4^ cells/well (equivalent to 2.0 × 10^5^ cells/mL) at a total volume of 100 mL and grown for 48 h until 80% confluence. Fresh cultures of the *C. perfringens* strains CP1, CP10, 25037-CP01 and CP22, grown overnight, were then measured by the OD at 600 nm, their concentrations adjusted to an OD of 1 and the cells loaded directly onto the CHIC-8E11 cells at a dose level of 50 mL/mL (CFU_loaded_). The plates were then centrifuged for 2 min at 3500× *g* and incubated for 1 h at 37 °C in conditions of 5% CO_2_. The cells were then washed five times with PBS and lysed with cold 0.1% Triton X-100 solution. The cell lysate suspensions were plated on TSB agar following a 10-fold serial dilution and the *C. perfringens* colonies counted (CFU_adhered_). The adhesion affinity of *C. perfringens* to the CHIC-8E11 cells was calculated according to the following formula:

*C. perfringens* adhesion (%) = [(CFU_adhered_)/(CFU_loaded_)] × 100 (1)

With probiotic CFS pre-treatment: The CHIC-8E11 cells were seeded in cell culture plates and grown to confluence as before. The culture media were then replaced with fresh media and CFS from *L. acidophilus* AG01 or *B. animalis* subsp. *lactis* AG02 (in each case, 3 mL/well, generated from 3 × 10^7^ CFU/mL) was added (CFU_loaded_) and the plates incubated overnight at 37 °C in 5% CO_2_. Cell culture medium without probiotic CFS served as the control. After incubation, the cells were washed with phosphate-buffered saline (PBS) before the addition of freshly cultured cells (CFU) of the *C. perfringens* strains CP1, CP10, 25037-CP01 or CP22 in culture medium, prepared as before and with the OD adjusted to 1, at a dose level of 50 mL/mL. The plates were centrifuged, incubated, washed, cells lysed and plated on TSB agar for *C. perfringens* colony counting (CFU_adhered_) and calculation of the percentage adhesion, as in the ‘without probiotic pre-treatment’ assay. The reduction in the percentage adhesion in the probiotic pre-treated treatments compared to the response in the medium control was calculated according to the following formula:

Reduction in *C. perfringens* adhesion (%) = [1 − (*C. perfringens* adhered_with probiotic CFS_/*C. perfringens* adhered_without probiotic CFS_)] × 100

### 2.5. Cell Permeability Assay

Without probiotic pre-treatment: The effect of the *C. perfringens* CFS and NetB toxin on the CHIC-8E11 cell permeability was assessed using the fluorescein isothiocyanate-dextran (FITC-D) permeability assay. Isolated CHIC-8E11 cells were seeded in 96-well cell culture plates fitted with permeable inserts (Thincert Pore 0.4 µm, Greiner Bio-one, Kremsmünster, Austria) at a density of 2.0 × 10^5^ cells per ml of complete cultivation medium (without phenol red), at a total volume of 100 μL. The cells were differentiated for 21 days into polarized epithelial cells (with serum at the apical surface of the cells and without serum at the basolateral surface). On day 7, the cells were moved to asymmetric conditions using the serum-free cultivation medium on the apical side and the complete medium on the basal side of the inserts. Differentiation was allowed to continue, refreshing the media every three days. The cells in the inserts were then challenged with the CFS from *C. perfringens* strains 25037-CP01 and CP22 (50 μL/mL), NetB toxin (1 μg/mL), or culture medium only (as a control) and incubated for a further 2 h at 37 °C in an atmosphere of 5% CO_2_. Thereafter, the medium in the apical side was removed, the cells were washed with PBS and 4 kDa FITC-dextran was added (FD4; 10 μg/mL), and the cells incubated for a further 3 h under the same conditions as before. The content of the basal compartment of the inserts was then collected and the fluorescence measured at 485/535 nm. An independent serial dilution of FD4 in complete medium was performed and an OD/FD4 standard curve produced to determine the amount of FD4 from the measurements of the OD. The cell permeability was expressed as the amount of FD4 (µg) in the basolateral compartment. 

With probiotic CFS pre-treatment: CHIC-8E11 cells were seeded in 96-well cell culture plates fitted with permeable inserts, differentiated for 21 days, and on d 7, moved to asymmetric conditions, as before. On day 20, the CFS from *L. acidophilus* AG01 or *B. animalis* subsp. *lactis* AG02 (3 µL, generated from 3 × 10^7^ CFU/mL) was added to the apical compartment of the cell culture inserts and the cells incubated at 37 °C in an atmosphere of 5% CO_2_ overnight. The cells in the culture inserts were then challenged with the CFS from *C. perfringens* strains 25037-CP01 or CP22, NetB toxin, or culture medium only (as a control) in the same way as described for the cell adhesion assay and incubated for a further 2 h under the same conditions as before. Thereafter, the FITC-dextran assay was performed as described for the cells without probiotic pre-treatment, the amount of FD4 in the apical and basal compartments of the wells was determined, and the percentage permeability calculated according to the following equation:

Permeability (%) = (FD4 content_basolateral_/FD4 content_apical_) × 100 (2)

The reduction in cell permeability in the probiotic CFS pre-treated wells compared to the control wells without probiotic CFS pre-treatment was then calculated according to the following formula:

Reduction in permeability (%) = [1 − (Permeability with probiotic CFS %/Permeability without probiotic CFS %)] × 100 (3)

### 2.6. Cytotoxicity Assay

First, the cytotoxicity of the CFS from the probiotics themselves (*L. acidophilus* AG01, *B. animalis* AG02) and of the CFS from *C. perfringens* 25037-CP01, *C. perfringens* CP22, and pure NetB toxin, directly on the CHIC-8E11 cells was investigated using the MTT assay. Then, the cytotoxicity induced by *C. perfringens* 25037-CP01, CP22, or NetB toxin when the CHIC-8E11 cells were pre-treated overnight with *L. acidophilus* AG01 or *B. animalis* AG02 CFS was investigated, and the percentage reduction in cytotoxicity calculated. The experimental details of the different treatments are described in Table 2. 

Without probiotic pre-treatment: The cytotoxicity of the CFS from *L. acidophilus* AG01, *B. animalis* AG02, Net B toxin, *C. perfringens* 25036-CP01 and *C. perfringens* CP22 against the CHIC-8E11 cells was assessed using the 3-(4,5-dimethylthiazol-2-yl)-2,5-diphenyltetrazolium cell viability assay (CyQUANT MTT^TM^ Cell Viability Assay). Isolated CHIC-8E11 cells were seeded in 96-well cell culture plates as before and grown to confluency. The culture media were then replaced with fresh media before introducing the CFS from *L. acidophilus* AG01, *B. animalis* AG02, *C. perfringens* strain CP1, CP10, 25037-CP01 or CP22 (all at 10, 20, 30, 40 and 50 µL/mL), or pure NetB toxin (added at 1 or 2 µg/mL), and the plates were then incubated for 4 h at 37 °C in an atmosphere of 5% CO_2_. Culture medium without *C. perfringens* CFS served as the control. After incubation, MTT stock solution was added and the assay procedure followed in accordance with the manufacturer’s instructions. The absorbance in the control and treated samples was read at 570 nm, where a higher absorbance indicated greater cell viability due to the known (pre-established) positive correlation between the absorbance and the percentage of non-viable cells. The cytotoxicity was expressed as the percentage absorbance in the treatment (test) wells compared to the control wells, according to the following formula:

Cytotoxicity % = [1 − (OD Test/OD control)] × 100. (4)

With probiotic CFS pre-treatment: CHIC-8E11 cells were seeded in 96-well cell culture plates and grown to confluency as before. The media were replaced with fresh media and probiotic CFS (*L. acidophilus* AG01 or *B. animalis* subsp. *lactis* AG02, according to the treatment) was added at a volume of 10, 20, 30, 40 or 50 µL/mL. The plates were then incubated overnight at 37 °C in an atmosphere of 5% CO_2_. Cells without the addition of probiotic CFS served as the control. After incubation, the CFS from *C. perfringens* strain 25037-CP01 or CP22 (30 µL/mL), or pure NetB toxin (1 µg/mL), was added. The plates were further incubated for 4 h under the same conditions as before. Thereafter, MTT stock solution was added, the assay procedure followed, the absorbance measured, and the percentage cytotoxicity calculated, as before. 

### 2.7. CHIC-8E11 Protein Expression without or with Probiotic CFS Pre-Treatment

Isolated CHIC-8E11 cells were seeded in 96-well cell culture plates fitted with permeable inserts and differentiated for 21 days as described for the cell permeability assay. On d 20, the CFS from *L. acidophilus* AG01 (3 mL, generated from 3 × 10^7^ CFU/mL) or *B. animalis* subsp. *lactis* AG02 (3 mL, generated from 3 × 10^7^ CFU/mL) was added to the apical side of each insert and the cells incubated overnight at 37 °C in an atmosphere of 5% CO_2_. Cells (CHIC-8E11) in culture medium without added probiotic CFS served as the control. After incubation, the cells were washed and then analyzed by 5% SDS solution. The lysate was collected and the expression of cytokines, chemokines and transcription factors measured by proteomics, as described by Shen et al. [34]. The sequence similarity to known chicken cell proteins was evaluated using the Chicken UniProt database, as described by Shen et al. [34]. 

### 2.8. Statistical Analysis

Data from all except the protein expression experiments were analyzed by the Kruskal–Wallis H test (one-way ANOVA on the ranks) to determine significant differences between groups. Differences were considered statistically significant at *p* < 0.05. For the protein expression data, a multivariate latent class model (LCA) was fitted to the proteomics manifestations, testing multiple pairwise treatment comparisons by the associated *t*-tests, controlling the false discovery rate (FDR) at a level of 5%. All the statistical analyses were conducted in Graph Pad Prism Software (version 9). 

## 3. Results 

### 3.1. Pathogenic Effects of C. perfringens against CHIC-8E11 Cells

**Figure 1:**
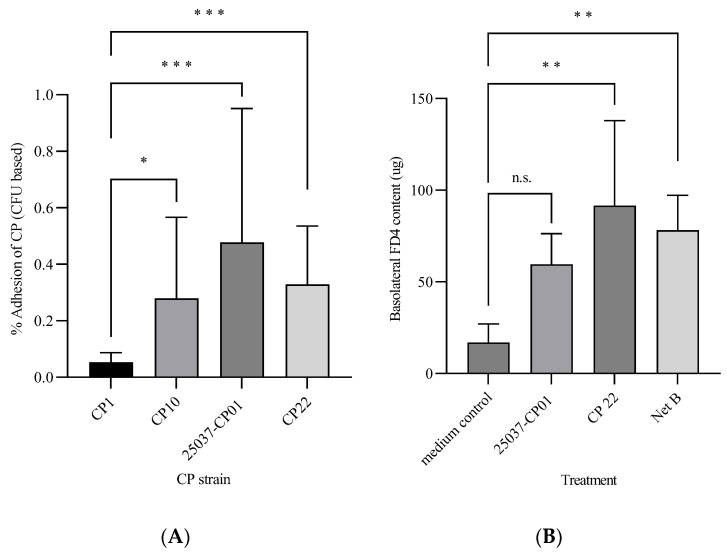
Adhesion of different strains of *C. perfringens* (as colony-forming units, CFUs) to CHIC-8E11 cells (**A**) and the effect of the CFS from different strains of *C. perfringens* on CHIC-8E11 cell permeability (**B**). (**A**) Adhesion was measured after 1 h at 37 °C in 5% CO_2_ and expressed as the percentage of CFUs adhered to CHIC-8E11 cells out of the total CFUs loaded onto cells at the start of the incubation. Experiments were performed three times with three replicates per experiment (nine replicates in total). (**B**) Permeability was measured using the fluorescein isothiocyanate-dextran (FITC-D) permeability assay and expressed as the amount (µg) of FD4 in the basolateral compartment of the cell culture inserts after 2 h incubation with *C. perfringens* CFS or NetB at 37 °C in an atmosphere of 5% CO_2_. Experiments were performed 3 times with 1–3 replicates per experiment (7 replicates in total). Values represent means ± SD; ***, **, *, statistically significantly different at *p* < 0.001, *p* < 0.01 and *p* < 0.1, respectively; n.s., non-significant at *p* < 0.05.

Cell adhesion: The percentage adhesion of *C. perfringens* cells (as the CFU) to CHIC-8E11 cells after 1 h of incubation is shown in Figure 1A. There was significant variation among the *C. perfringens* strains; the percentage adhesion was greater for 25037-CP01, CP22 and CP10 than for CP1 (respectively, by 0.42, 0.28 and 0.22% points; *p* < 0.05).

Cell permeability: Incubation of CHIC-8E11 cells with the CFS from *C. perfringens* strain CP22 or with pure NetB increased the FD4 content (µg) of the basolateral compartment of the cell culture inserts (indicating increased cell permeability) compared with the control (91.6 ± 17.5 µg in CP22 and 78.15 ± 19.01 µg in NetB, vs. 16.9 ± 3.8 µg in the control; *p* < 0.01; Figure 1B). Challenge with the CFS from strain 25037-CP01 numerically but not statistically significantly increased the basolateral FD4 content (59.6 ± 10.2 µg in treatment 25037-CP01 vs. 16.9 ± 3.8 µg in the control).

**Figure 2:**
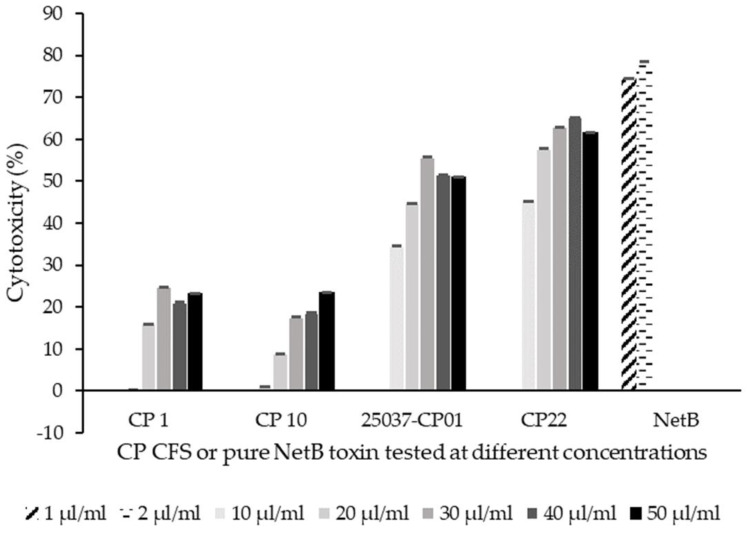
Effect of the cell-free supernatant (CFS) from *C. perfringens* (CP) strains and NetB on CHIC-8E11 cell viability. Cell viability was measured by the 3-(4,5-dimethylthiazol-2-yl)-2,5-diphenyltetrazolium cell viability assay after 4 h incubation with *C. perfringens* CFS or NetB at 37 °C in an atmosphere of 5% CO_2_. Results are expressed as the cytotoxicity percentage compared with the control treatment that contained cell culture medium but no CFS or NetB. Experiments were performed three times with two or three replicates per treatment (eight replicates in total). Values represent means ± SD. Differences between treatments were non-significant at *p* < 0.05.

Cell viability: Cell-free supernatant from all the *C. perfringens* strains (CP1, CP10, 25037-CP01 and CP22) and pure NetB toxin induced the cytotoxicity of CHIC-8E11 cells after 4 h incubation (Figure 2). The effects were dose- and strain-dependent. Pure NetB toxin added at 1 or 2 µg/mL had the greatest cytotoxic effect (75–70% cytotoxicity after 4 h; NetB was not administered above 2 µg/mL because of its observed strong cytotoxic effect), followed by the CFS from NetB-positive CP22 (45–62% cytotoxicity across dose levels of 10–50 µL/mL), NetB-positive 25037-CP01 (35–56% cytotoxicity), NetB-negative CP10 (1–24% cytotoxicity) and NetB-negative CP1 (0.3–25% cytotoxicity). The dose-response effect plateaued above 30 µL/mL for all the strains except CP10. 

### 3.2. Effect of Pre-Treatment with Probiotics on C. perfringens Pathogenic Effects

**Figure 3:**
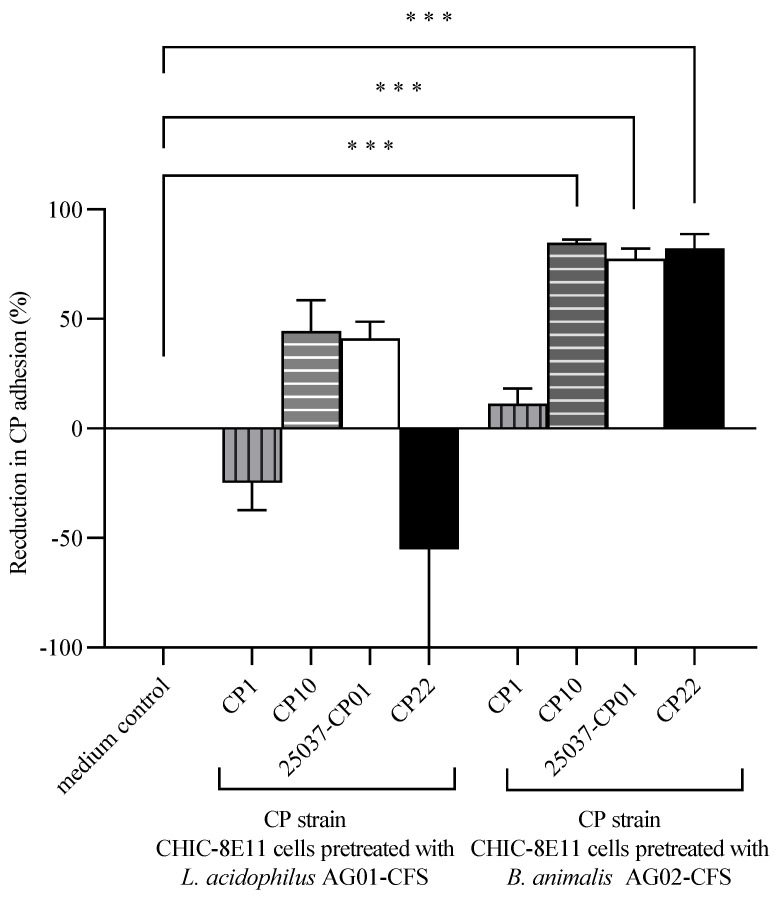
Percentage reduction in the adhesion of *C. perfringens* (CP) to CHIC-8E11 cells following pre-treatment with probiotic cell-free supernatant (CFS). The reduction in the adhesion percentage in the probiotic CFS pre-treated groups was compared to the response in the medium control. Experiments were performed three times with three replicates per experiment (nine replicates in total). Values represent means ± SD. ***, statistically significant at *p* < 0.001.

Cell adhesion: Overnight pre-treatment of CHIC-8E11 cells with the CFS from probiotic *B. animalis* subsp. *lactis* AG02 markedly reduced the cell adhesion of the *C. perfringens* strains CP10, 25037-CP01 and CP22 (respectively, by 84.8 ± 3.7, 77.4 ± 14.0, and 82.3 ± 15.6, vs. control; *p* < 0.001), but it had no significant effect on the CP1 adhesion (Figure 3). Pre-treatment of CHIC-8E11 cells with the CFS from probiotic *L. acidophilus* AG01 had no statistically significant effect on the cell adhesion by any *C. perfringens* strain. 

**Figure 4:**
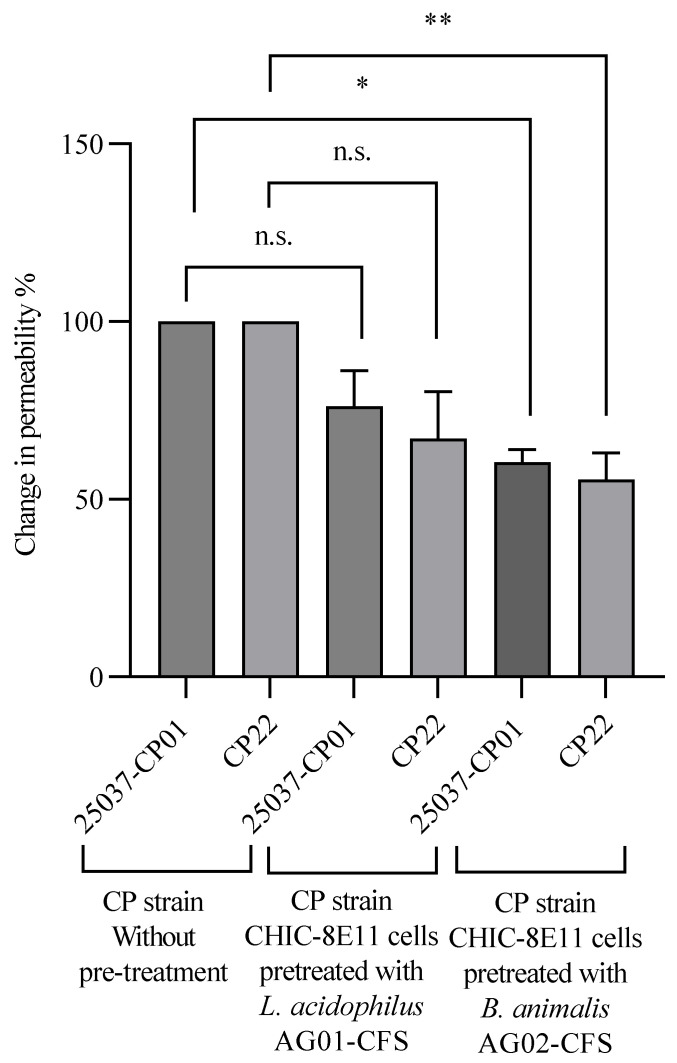
Permeability change in CHIC-8E11 cells challenged with *C. perfringens* cell-free supernatant (CFS) following pre-treatment with probiotic CFS. Permeability was measured using the fluorescein isothiocyanate-dextran (FITC-D) permeability assay and is expressed as a percentage of the amount (μg) of FD4 in the basolateral compartment of cell culture inserts relative to that in the apical compartment after 2 h incubation with *C. perfringens* CFS. The reduction in permeability (%) was calculated relative to the medium control (without probiotic CFS pre-treatment). Experiments were performed 3 times with 1–3 replicates per experiment (5 replicates in total). Values represent means ± SD. *, statistically significant at *p* < 0.05; **, statistically significant at *p* < 0.01; n.s., non-significant at *p* < 0.05.

Cell permeability: Overnight treatment of CHIC-8E11 cells with the CFS (30 µL/mL) from *L. acidophilus* AG01 had no significant effect on the cell permeability as compared to the control, while the CFS from *B. animalis* subsp. *lactis* AG02 significantly improved (*p* < 0.01) the enterocyte barrier (Supplementary Materials, Figure S1). Overnight pre-treatment of CHIC-8E11 cells with the CFS from *B. animalis* subsp. *lactis* AG02 significantly reduced the increase in the permeability of cells challenged with the CFS from *C. perfringens* strains 25037-CP01 or CP22 (respectively, by 40% points (*p* < 0.05) and by 44% points (*p* < 0.01), vs. no probiotic preculture; Figure 4). The increase in the permeability of cells challenged with the CFS from strain 25037-CP01 or CP22 was numerically but not statistically significantly reduced by pre-treatment of the cells with the CFS from probiotic *L. acidophilus* AG01. 

Cell viability: The effect of the various treatments on the viability of CHIC-8E11 cells (as indicated by the % cytotoxicity) is shown in Table 3. The culture medium (medium control) had no effect on the cell viability. Overnight treatment of the CHIC-8E11 cells with 10 to 50 µL/mL CFS from *L. acidophilus* AG01 or *B. animalis* AG02 induced cytotoxicity at a relatively low level, of 11.17% (±0.28) with *L. acidophilus* AGO1 CFS administered at 50 µL/mL and 9.52% ± 0.28) with *B. animalis* AG02 CFS administered at 30 µL/mL of *B. animalis* AG02 CFS. In contrast, incubation of CHIC-8E11 cells with *C. perfringens* 25037-CP01 or CP22 CFS at the lowest dose (10 µL/mL) induced cytotoxicity at a level of 34.57% (±0.06) and 45.22% (±0.11), respectively, whereas pure NetB toxin applied at 1 µg/mL induced cytotoxicity at a level of 74.60% (±0.04).

Overnight pre-treatment of CHIC-8E11 cells with the CFS from *L. acidophilus* AG01 or *B. animalis* subsp. *lactis* AG02, prior to challenge for 4 h with the CFS from *C. perfringens* strains 25037-CP01 or CP22, or with pure NetB toxin, numerically but not significantly reduced the cytotoxicity percentage compared with the control treatment (Table 3). The magnitude of the reduction appeared to be dependent on the concentration of the probiotic CFS applied (greater with an increasing concentration) and differed between the two probiotic strains. At the lowest probiotic CFS concentration (10 µL/mL), there were no reductions in the cytotoxicity of the *C. perfringens* CFS from strains 25037-CP01 or CP22 against CHIC-8E11 cells, but there was a numerical reduction in the cytotoxicity of NetB (−4% and −20%, vs. the control, for cells pre-treated with CFS from *L. acidophilus* AG01 and *B. animalis* subsp. *lactis* AG02, respectively). At the highest probiotic CFS concentration (50 µL/mL), both probiotic strains numerically reduced the cytotoxicity of the CFS from strains 25037-CP01, and CP22, and of NetB against CHIC-8E11 cells but with differing efficacy: the CFS from *B. animalis* subsp. *lactis* AG02 was numerically more effective than that from *L. acidophilus* AG01 at reducing the cytotoxicity of CP22 (−21% vs. 16%, respectively) and NetB (−34% vs. −18%, respectively), whereas the two probiotics were equally effective at reducing the cytotoxicity of strain 25037-CP01 (−24% and −24%, respectively, vs. control). 

**Figure 5:**
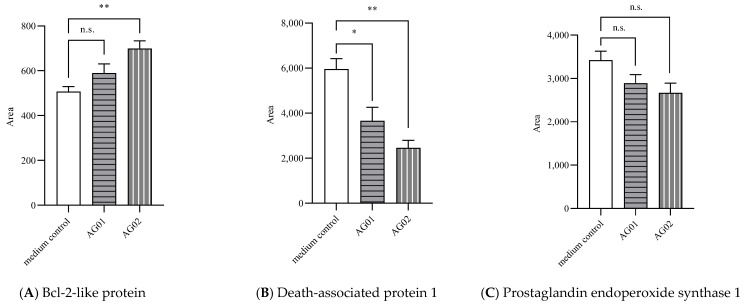

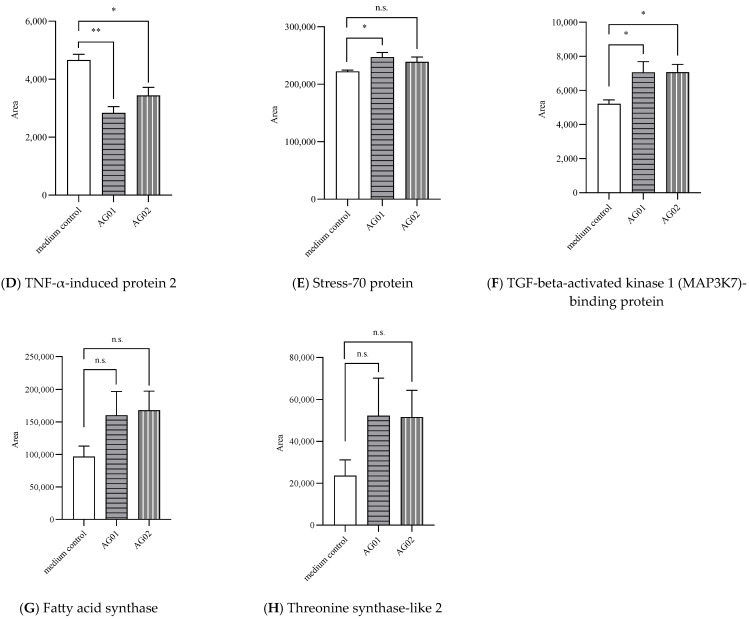
(**A**–**H**) Effect of the pre-treatment of CHIC-8E11 cells with probiotic CFS on the expression of cell proteins. Experiments were performed three times with two replicates per treatment (six replicates in total). Data represent means ± SD. * statistically significant at *p* < 0.05; **, statistically significant at *p* < 0.01; n.s., non-significant at *p* < 0.05.

Protein expression: Compared to the control treatment, the CFS from probiotic *B. animalis* subsp. *lactis* AG02 significantly upregulated the CHIC-8E11 expression of Bcl-2-like protein (+38% area under the curve (AUC) vs. control, *p* < 0.05; Figure 5A), whereas the CFS from *L. acidophilus* AG01 upregulated the expression of stress-70 protein (+11% AUC vs. control, *p* < 0.05; Figure 5E). Both upregulated the expression of TGF-beta activated kinase 1/MAP3K7-binding protein 2 (+36% and +36% AUC for *L. acidophilus* AG01 and *B. animalis* subsp. *lactis* AG02, respectively, vs. control, *p* < 0.05; Figure 5F) and downregulated the expression of death-associated protein 1 (−38% and −59% AUC for *L. acidophilus* AG01 and *B. animalis* subsp. *lactis* AG02, respectively, vs. control, *p* < 0.05; Figure 5B) and TNF-α-induced protein 2 (−39% and −26% AUC for *L. acidophilus* AG01 and *B. animalis* subsp. *lactis* AG02, respectively, vs. control, *p* < 0.05; Figure 5D). The expressions of prostaglandin endoperoxide synthase 1 (Figure 5C), fatty acid synthase (Figure 5G) and threonine synthase-like 2 (Figure 5H) were not significantly affected by the tested CFSs.

## 4. Discussion

The need for effective alternatives to antibiotics in feed is being driven by their prohibition in many regions of the world, by changes in consumer preferences, and by growth of the ‘one health’ concept, which recognizes and supports the inter-relationships between human, animal and environmental health. Studying probiotics as potential alternatives to antibiotics for controlling and reducing *C. perfringens* pathogenicity in the context of NE requires a representative model of poultry intestinal responses. As recently highlighted by Marks et al. [37], a reliable, standardized, chicken enterocyte cell model (such as the CHIC-8E11 cell line model) would offer a more representative means of studying in vitro host–pathogen interactions than existing human or porcine cell models, but it requires further study. The present study aimed to use the novel CHIC-8E11 cell line to characterize the key pathogenic traits of *C. perfringens* (cell adhesion, disruption of intestinal barrier integrity and cytotoxicity) and then to evaluate the modulating effect of two probiotic strains on those traits. In performing this research, it was recognized that our knowledge of the characteristics of the CHIC-8E11 cell line and its applicability to the study of pathogen–host cell interactions is still developing and that further studies will be needed to fully assess its merits and limitations. On the one hand, the cell line has been relatively well characterized, including its expression of multiple elementary cell components (epithelial cells specialized for nutrient absorption (enterocytes), antimicrobial peptide secretion (Paneth cells) or mucus secretion (goblet cells)) as well as physiological resemblance (stem cells and Paneth cells are located at the base of epithelial crypts while the others face toward the lumen). On the other hand, under experimental conditions, the cells are flat, stretched and equally exposed to media, CFS and bacteria, which does not fully replicate what would happen in vivo, where any combination of exposure scenarios could occur. Thus, it is important not to over-extrapolate the results to the in vivo situation. Some authors have attempted to overcome the limitations of a 2-dimensional cell line model by using 3D animal models such as intestinal organoid models [38]. Avian mini gut models or ‘enteroïds’ have been developed as a model to study host–pathogen interactions [39] and used to study the effect of infection of the epithelial apical surface with *Salmonella Typhimurium*, *influenza* A virus and *Eimeria tenella*. The findings of the present study could usefully be compared with those from the application of *C. perfringens* with and without probiotic pre-treatment to the enteroïd model to further validate the applicability of the CHIC-8E11 model. 

Following the preliminary work that confirmed the CHIC-8E11 cell line was of chicken origin and exhibited the expected structural complexity of the intestinal epithelium, the subsequent experiments focused on evaluating the CHIC-8E11 cell responses to three key pathogenic traits of *C. perfringens* implicated in NE: (1) the adhesion to intestinal epithelial cells that is the critical first step in bacterial colonization and cell invasion [40] and that induces the rapid upregulation of toxin production that increases subsequent pathogenicity [41]; (2) the reduction in cell permeability that reduces the gut barrier integrity, potentially allowing the entry and systemic spread of *C. perfringens* and secreted toxins [42]; and (3) the reduction in cell viability that results in damage to the epithelial layer, which is the critical barrier regulating the passage of nutrients across the epithelium and preventing bacterial infiltration of the intestinal lining. 

The cell adhesion assay results demonstrated that all four *C. perfringens* strains were capable of adhering to CHIC-8E11 cells, albeit it at a low level (<1% across all the treatments). The percentage values reported in the literature vary widely across studies and pathogen strains, but the values reported here fall within that range and are therefore not unusual. For example, Trejo et al. [43] reported adhesion percentages of 0.5–2% across treatments for UV-treated and non-irradiated strains of *Clostridium difficile* to human Caco-2/TC7 cells. The protocol followed in our study involved a relatively short period of contact between the pathogen CFU and CHIC-8E11 cells (6 h) compared to some other studies in the literature that have used 12 h. This may also have given rise to the low level of response. The washing of the cells five times prior to centrifugation was expected to have removed any unadhered bacteria from the experimental sample (and to have selected only those that were strongly attached or that had invaded the CHIC-8E11 cells). Thus, the subsequent centrifugation step was not expected to have effected any significant change in the percentage adhesion. However, this was not tested. Therefore, the differences in the adhesion percentage between the treatments should be interpreted as relative rather than absolute differences. The comparison between the treatments with and without probiotic treatment was of more interest in this analysis than the capability of CP to adhere to CHIC-8E11 cells per se. Nevertheless, the results indicated that the affinity of attachment varied significantly among the strains. The variation in the adhesion percentage suggests variation in the relative virulence of the different *C. perfringens* strains and the higher percentage adhesion of NetB-positive strains 25037-CP01 and CP22 relative to NetB-negative strain CP1 implicates NetB toxin, or another factor associated with NetB-positive strains, as being important for adhesion. In vivo, bacterial adhesion is thought to be mediated by the combined action of fimbriae appendages, flagella and biofilms produced on the bacterial cell surface [44,45]. Recent studies by Lepp et al. [46,47] have implicated a specific domain present within the genome of 68–85% of NetB-positive strains of *C. perfringens* isolated from poultry (that is absent from the majority of NetB-negative strains) that encodes an adhesive pilus, which binds to collagen in vitro and participates in adherence. This would explain the higher adhesion of the NetB+ strains in the present study. Other toxins secreted by the strains 25037-CP01 and CP22, both of which are G strains, could also have aided in the adhesion process, although this was not evaluated. For example, the toxin TpeL that is produced by type A and G strains has been implicated in the adhesion process of pathogenic *C. perfringens* [40] to host cells.

It is well known that the intestinal barrier is disrupted in both clinical and subclinical NE, the latter of which may develop during another gastrointestinal infection (for example, that caused by *Eimeria* parasites) in the presence of *C. perfringens*. Latorre et al. and Akerele et al. both observed increased gut permeability (increased levels of serum FITC-dextran) in broilers following *C. perfringens* and/or *Eimeria* challenge [42,48]. The effector molecules and their modes of action are not fully understood. However, in vitro and ex vivo studies have shown that a variety of *C. perfringens*-secreted toxins, including enterotoxin (CPE) [49], epsilon toxin [50] and delta-toxin [51,52], can interact with tight junctions in the epithelium that control the diffusion of substances across the paracellular space. These interactions impair the functionality of the tight junctions, leading to increased epithelial permeability. In the present study, the CFS from *C. perfringens* strain CP22 significantly increased the permeability of the CHIC-8E11 cells after 2 h incubation, consistent with the literature suggesting that substances within the CFS secreted by the pathogen disrupted the barrier integrity. The specific nature of these substances cannot be confirmed, but the 3.6-fold increase (above the control) in the basolateral FD4 content (above control) in the NetB-positive CP22 treatment implicates NetB in this effect. Although the CFS from the other NetB-positive strain (25037-CP01) did not statistically significantly increase cell permeability, the trend in the data was in the same direction. To the best of the authors’ knowledge, a direct effect of NetB toxin on chicken intestinal cell permeability (or on that of intestinal cells from other species models) has not previously been reported. Whether tight junction proteins were a target for NetB in this effect is unknown; increased IL-10 production has been implicated as a response of CHIC-8E11 cells to challenge with *C. perfringens*-derived NetB and α-toxin [53] or with *Campylobacter jejuni* [54] but was not evident (as assessed by PCR [54].

The cell viability assay results demonstrated that the CHIC-8E11 cells were permissive to the CFS from pathogenic *C. perfringens* strains. All the strains resulted in measurable cytotoxicity after 4 h of incubation, with marked variation among the strains and dose levels in the potency of effect. It is hypothesized that the strain effect could have been linked to the specific toxins present in the CFS and to their relative concentrations. Specifically, the very high, apparently dose-related, cytotoxic effect of pure NetB (>70% cytotoxicity against CHIC-8E11 cells when administered at 1 or 2 μL/mL) implicates it as a causative agent in the observed cytotoxicity of the NetB-positive 25037-CP01 and CP22. This is consistent with the literature describing NetB as having a direct cytotoxic effect on poultry intestinal cells in NE that is attributable to its induction of pore formation in the cell membrane, which results in cell leakage and apoptosis [7]. Indeed, Keyburn et al. showed that a NetB mutant of *C. perfringens* was unable to induce gut lesions in infected poultry, directly implicating NetB in the pathogenic effects of *C. perfringens* on poultry intestinal cells [7]. For the cytotoxic effect to be mediated, the NetB toxin must initially bind to a receptor located on the cell membrane [55], which in the present in vitro simulation will have been limited by the number of cells present in the incubation. The plateauing of the cytotoxicity of the CFS from 25037-CP01 and CP22 above 30 mL/mL could therefore reflect the saturation of receptor sites within the available cells present in each well. Meanwhile, the lower cytotoxic effect of the CFS from the NetB-negative, a-positive, strains CP1 and CP10 could have been due to the presence of a-toxin in the CFS; a-toxin is well established as a major causal factor of NE in poultry, causing cell lysis by the hydrolysis of phosphatidylcholine and sphingomyelin in the cell membrane [56]. Other (unidentified) toxins and active substances present in the CFS could also have contributed to the observed cytotoxic effects of the tested strains; *C. perfringens* secretes 18 or more different toxins as well as bacteriocins and hydrolytic enzymes, all of which contribute to its virulence and pathogenicity in NE in vivo [57]. 

In the second part of the study, in which the effect of the CFS from the probiotic strains on the CHIC-8E11 response to *C. perfringens* challenge was evaluated, several beneficial and complimentary effects were observed. It is acknowledged that these effects must be interpreted with some caution because, whilst the percentage changes were sizeable and sometimes significant, the absolute values (for example, from the adhesion assay) were often low and therefore the biological relevance of the percentage changes is unknown. Nevertheless, we considered that comparing the relative (%) differences between probiotic pre-treated and untreated groups may generate useful insights into the mode of effect of the probiotics for further study in in vivo trials. The marked and significant reduction in the adhesion of three out of four *C. perfringens* strains (CP10, 25037-CP01 and CP22) to the CHIC-8E11 cells following their pre-treatment with the CFS from *B. animalis* subsp. *lactis* AG02, and the numerical reduction in the adhesion of two out of four *C. perfringens* strains (CP10 and 25037-CP01) following their pre-treatment with the CFS from *L. acidophilus* AG01, suggests the presence of beneficial effector molecule(s) in the CFS from both probiotics that disrupted or blocked adhesion. The differing responses of the different strains suggest phenotypic diversity in the susceptibility of different *C. perfringens* strains to effector molecules present in the CFS. This was previously observed by Garde et al., who reported strain-dependent growth inhibition of *C. perfringens* in response to metabolites secreted by lactic acid bacteria with antimicrobial activity [58]. This pathogen diversity underlines the need to select probiotics with a wide spectrum of efficacy and/or to select multiple probiotics with complementary activity to achieve a consistently beneficial effect across varied settings in the field. The efficacy of the two probiotic strains tested herein against three out of four *C. perfringens* strains is promising in this regard. 

The cell-free supernatant from *B. animalis* subsp. *lactis* AG02, but not from *L. acidophilus* AG01, significantly reduced the negative effect of *C. perfringens* CFS on CHIC-8E11 cell permeability and was equally effective against the strains 25037-CP01 and CP22 (40–44% reduction achieved across both strains). The duration of this beneficial effect is unknown. However, this is not so relevant for the in vivo situation, where repeated application of the probiotics via the regular consumption of feed would be expected to extend the duration of the beneficial effect. Given the critical role of epithelial barrier integrity in regulating the passage of substances into the body from the gut lumen, and the knowledge that this permeability is impaired in NE [42], an improvement in barrier integrity due to the CFS from *B. animalis* subsp. *lactis* AG02 would be expected to reduce toxin and bacterial translocation and *C. perfringens* pathogenesis. Indeed, in vivo supplementation of *C. perfringens*-challenged birds with a combination of the two probiotics (*L. acidophilus* AG01 and *B. animalis* subsp. *lactis* AG02) has been observed to reduce the lesion score back to the level of the control treatment without pathogen [26]. Beneficial effects on broiler gut barrier integrity have also been observed for other probiotic species: authors showed that dietary supplementation with *L. plantarum* reduced serum FITC-dextran absorption, indicating a positive impact on gut permeability in (unchallenged) broilers [59], whilst Zhao et al. [60] observed upregulated expression of tight junction proteins in *C. perfringens*-challenged broilers supplemented with probiotic *B. licheniformis* H2. In vitro, Al-Sadi et al. demonstrated that a strain of *B. bifidum* improved epithelial integrity, and in a live mouse model, this was found to be due to a tightening of the intestinal tight junctions that was linked to the attachment of the bacterium to the Toll-like receptor 2 (TLR-2) complex on the apical surface of the enterocyte membrane [61]. Studies of other probiotic genera have similarly implicated tight junctions as a key target for probiotic effects on gut barrier integrity [62,63]. 

The measured cytotoxic effect of the CFS from the probiotics themselves on CHIC-8E11 cells was low (less than 10%), suggesting that the cell permeability measurements were measuring permeability and not cytotoxicity, which might otherwise allow dextran to diffuse across the cell membrane and be recorded as a change in permeability. The beneficial effects of the probiotics on the *C. perfringens*-induced effects on cell permeability and viability were achieved by pre-treatment with the CFS rather than the intact organism. This suggests that the effects were mediated via effector molecules within the CFS rather than via binding of the probiotic to a cell surface receptor. *Bifidobacterium sp.* can produce a variety of antimicrobial compounds, including short-chain fatty acids, hydrogen peroxide, nitric oxide and bacteriocins [64], any of which could affect the outer membrane of *C. perfringens* cells and/or its capacity to adhere to host cells. The protein expression results may also provide some insight here: both BCL-2-like protein and TAB2 (TGF-β-activated kinase 1 (Map3K7)-binding protein) were upregulated and TNF-a-induced protein 2 was downregulated in the CHIC-8E11 cells following their pre-treatment with the CFS from *B. animalis* subsp. *lactis* AG02 (but not by pre-treatment with the CFS from *L. acidophilus* AG01). The Bcl-2-like protein is an anti-apoptotic protein that protects the permeabilization of the outer membrane to preserve its integrity [65], so its upregulation could have served to reduce epithelial permeability, whereas TAB2 is involved in IL-1 signaling, which regulates inflammation through the NF-kB pathway [66]. 

The probiotic CFS increased the expression of proteins involved in the anti-inflammatory TGF-beta pathway. Meanwhile, certain pro-inflammatory cytokine pathways were downregulated (prostaglandin endoperoxide and TNF-α). In addition, the results suggested that the probiotic CFS may have enhanced epithelial cell viability via increased expression of the BCL-2 and stress-70 proteins and the inhibition of apoptosis death-associated protein 1. An imbalance between pro-inflammatory and anti-inflammatory cytokines results in disease progression and tissue damage and limits the resolution of intestinal inflammation. It has been well documented that *C. perfringens* infection induces inflammatory cytokine production in the intestine of broilers [67]. Based on the collective results, it is hypothesized that the pre-treatment of CHIC-8E11 cells with the probiotic CFS protected the cells from pathogenic inflammation caused by *C. perfringens* via establishing a balance between immunity and tolerance. Further studies are needed to test this hypothesis.

The effect of the CFS from each of the probiotic strains on *C. perfringens*-induced cytotoxicity suggests that both probiotics exerted independent beneficial effects. However, the absence of statistical significance when compared to the response of the control treatment limits the ability to draw firm conclusions. The numerical reductions in cytotoxicity following probiotic pre-treatment were 15 to 25% when applied at the maximum dose level of 50 µL/mL. The (numerically) differential degree of effect of the probiotics against the individual *C. perfringens* strains may suggest a complementarity between the two strains. A beneficial effect of *L. acidophilus* AG01 CFS in reducing *C. perfringens* cytotoxicity would be consistent with the recent findings of Guo et al., in which the CFS from *Lactobacilli* cultures suppressed the growth of *C. perfringens* after 24 h of incubation and significantly reduced their cytotoxic effect against chicken intestinal epithelial cells (as indicated by measurements of the lactate dehydrogenase release) [68]. Similarly, a beneficial effect of *B. animalis* AG02 CFS in reducing *C. perfringens*-induced cytotoxicity would be consistent with the findings of Shanmugasundaram et al., who observed that *B. animalis* CFS reduced the proliferation of *C. perfringens* after 24 h of incubation in a positive dose-dependent manner [23]. Guo et al. showed that the beneficial effect of *Lactobacillus* sp. CFS was mediated via a decrease in the production of a-toxin as well as by direct degradation of the a-toxin by the probiotic CFS [67]. This mode of action could be equally relevant to the present study, given that both tested *C. perfringens* strains (CP22 and 25037-CP01) were a-toxin-positive strains. The observation that the CFSs from both probiotics were also effective at (numerically) reducing the cytotoxicity induced by administration to cells of the NetB toxin itself additionally suggests a mode of action targeted at NetB. Further studies are needed to confirm this. 

## 5. Conclusions

This study represents the first report of the use of an intestinal epithelial chicken cell model, CHIC-8E11, to study key pathogenic traits of *C. perfringens* and to assess the potential of two probiotic strains to mitigate those effects. There are some limitations associated with the use of the in vitro cell line model, which is two-dimensional and cannot fully replicate the complexity of the in vivo gut architecture and physiology, and also with the interpretation of our results, where the response levels were low in absolute terms, so that the biological significance of the percentage differences between the treatments cannot be established (e.g., in the cell adhesion assays). However, in general, the present results confirm the suitability of the cell line for future studies of *C. perfringens* pathogen–host cell interactions in the context of NE and suggest that it could be useful for the screening of probiotics and development of approaches for the prevention of NE, in the same way that human cell line models have proved invaluable for the development of human medicines [69]. 

All four tested *C. perfringens* strains were found capable of adhering to CHIC-8E11 cells, with varying degrees of affinity; the CFS from one strain (CP22) increased the permeability of the CHIC-8E11 cells, with NetB toxin implicated in this effect, and the CFSs from all the strains were cytotoxic against CHIC-8E11 cells in a dose-dependent manner that varied across the strains and was apparently linked to the NetB content. The pre-treatment of CHIC-8E11 cells with the CFS from *L. acidophilus* AG01 numerically reduced the adhesion of two out of four *C. perfringens* strains to the CHIC-8E11 cells, whereas pre-treatment of CHIC-8E11 cells with the CFS from *B. animalis* subsp. *lactis* AG02 markedly and significantly reduced the adhesion of three out of four *C. perfringens* strains to the CHIC-8E11 cells and reduced the negative effect of the two NetB-positive strains on the cell permeability. In addition, the CFS from either probiotic was effective in reducing the *C. perfringens*-induced cytotoxicity and appeared to exhibit a complementarity of effect against the individual strains. These results support the combination of *L. acidophilus* AG01 and *B. animalis* subsp. *lactis* AG02 in future in vitro and in vivo assays to further assess their potential as alternatives to antibiotics for the prevention and control of NE in broilers. 

## Figures and Tables

**Table 1:** Expression of genes encoding virulence factors by *C. perfringens* strains used in the study.

*C. perfringens* Strain No.	Country of Origin and Year of Isolation	Expression of Gene Encoding Virulence Factor	
		α Toxin	NetB Toxin
CP1	UK—2017	+	-
CP10	SWE—2018	+	-
25037-CP01	USA—2016	+	+
CP22	USA—2016	+	+

**Table 2:** Experimental details of the different treatments tested in the cytotoxicity assay.

Treatment No.	Treatment Details	Data Expressed as: C, % Cytotoxicity, or RC, % Reduction in Cytotoxicity
Baseline treatments; CHIC-8E11 cells treated with…		
1	Medium control (at 10, 20, 30, 40 and 50 µL/mL)	C
2	CFS *L. acidophilus* AG01 (at 10, 20, 30, 40 and 50 µL/mL)	C
3	CFS *B. animalis* subsp. *lactis* AG02 (at 10, 20, 30, 40 and 50 µL/mL)	C
4	CFS 25037-CP01 (at 10, 20, 30, 40 and 50 µL/mL)	C
5	CFS CP22 (at 10, 20, 30, 40 and 50 µL/mL)	C
6	NetB toxin (at 1 µg/mL)	C
Experimental treatments; CHIC-8E11 cells treated with…		
7	CFS *L. acidophilus* AG01 (at 10, 20, 30, 40 and 50 µL/mL) overnight pre-treatment, followed by 4 h contact time with 30 µL/mL of 25037-CP01 CFS	RC vs. T4 (at 30 µL/mL)
8	CFS *L. acidophilus* AG01 (at 10, 20, 30, 40 and 50 µL/mL) overnight pre-treatment, followed by 4 h contact time with 30 µL/mL of CP22 CFS	RC vs. T5 (at 30 µL/mL)
9	CFS *B. animalis* AG02 (at 10, 20, 30, 40 and 50 µL/mL) overnight pre-treatment followed by 4 h contact time with 30 µL/mL of 25037-CP01 CFS	RC vs. T4 (at 30 µL/mL)
10	CFS *B. animalis* AG02 (at 10, 20, 30, 40 and 50 µL/mL) overnight pre-treatment, followed by 4 h contact time with 30 µL/mL of CP22 CFS	RC vs. T5 (at 30 µL/mL)
11	CFS *L. acidophilus* AG01 (at 10, 20, 30, 40 and 50 µL/mL) overnight pre-treatment, followed by 4 h contact time with 1 µL/mL of NetB toxin	RC vs. T6 (at 1 µL/mL)
12	CFS *B. animalis* AG02 (at 10, 20, 30, 40 and 50 µL/mL) overnight pre-treatment, followed by 4 h contact time with 31 µL/mL of NetB toxin	RC vs. T6 (at 1 µL/mL)

**Table 3:** Percentage cytotoxicity of CHIC-8E11 cells induced by the CFS from probiotic *L. acidophilus* AG01, probiotic *B. animalis* AG02, *C. perfringens* strain 25036-CP01, *C. perfringens* strain CP22, or NetB toxin, and the percentage reduction in cytotoxicity induced by *C. perfringens* or NetB toxin when the CHIC-8E11 cells were pre-treated overnight with *L. acidophilus* AG01 or *B. animalis* AG02 CFS. Cell viability was evaluated by the 3-(4,5-dimethylthiazol-2-yl)-2,5-diphenyltetrazolium cell viability assay. Experiments were performed three times with two or three replicates per experiment (eight replicates in total). Values represent means (±SD). Differences between treatments were not statistically significantly different at *p* < 0.05.

Response Measure	Treatment No.	Dose of Application to CHIC-8E11 Cells					
		1 µg/mL	10 µL/mL	20 µL/mL	30 µL/mL	40 µL/mL	50 µL/mL
Cytotoxicity (%) of baseline treatments (±SD)	1	nd	0 (0.04)	0 (0.04)	0 (0.04)	0 (0.04)	0 (0.06)
	2	nd	1.12 (0.05)	6.65 (0.17)	4.64 (0.06)	10.3 (0.09)	11.17 (0.28)
	3	nd	7.78 (0.28)	7 (0.41)	9.52 (0.28)	6.2 (0.32)	7.26 (0.35)
	4	nd	34.57 (0.06)	44.73 (0.03)	55.77 (0.04)	51.71 (0.03)	51.15 (0.02)
	5	nd	45.22 (0.11)	57.78 (0.02)	63.04 (0.05)	65.19 (0.12)	61.70 (0.02)
	6	74.60 (0.04)	nd	nd	nd	nd	nd
Reduction in cytotoxicity (%) induced by probiotic pre-treatment vs. baseline treatment ^1^ (±SD)	7	nd	−7.75 (0.02)	−1.47 (0.16)	13.31 (3.59)	15.50 (2.02)	24.39 (3.37)
	8	nd	−3.71 (0.67)	16.85 (2.50)	9.01 (0.34)	13.48 (2.54)	24.42 (4.75)
	9	nd	−8.45 (0.69)	2.15 (0.04)	5.72 (0.79)	13.84 (5.40)	16.07 (2.40)
	10	nd	−19.92 (0.18)	0.78 (0.37)	10.78 (0.02)	19.60 (0.3)	21.23 (0.33)
	11	nd	4.10 (0.25)	5.21 (0.59)	17.80 (1.36)	10.56 (1.07)	15.50 (0.005)
	12	nd	20.24 (2.08)	23.02 (1.02)	26.70 (3.32)	29.22 (0.20)	33.76 (3.75)

^1^ Details of the comparator treatments in each case are given in Table 2. nd, not determined.

## Data Availability

The datasets generated in this study are available upon request from the corresponding author.

## Supplementary Materials

The following supporting information can be downloaded at https://www.mdpi.com/article/10.3390/microorganisms12040839/s1, Figure S1: Effect of the CFS from two probiotic strains, *L. acidophilus* AG01 and *B. animalis* AG02 (30 µL/mL), on the CHIC-8E11 cell permeability; Table S1: Proteins, TaqMan ID numbers and expression results for the CHIC 8E11 cell line verification.

## Abbreviations

AUC	Area under the curve
CFU	Colony-forming unit
CP	*Clostridium perfringens*
CFS	Cell-free supernatant
d	Day(s)
FBS	Fetal bovine serum
FD4	FITC-dextran (4 KD)
LCA	Latent class model
MRS	Man–Rogosa–Sharpe broth
MTT	3-(4,5-dimethylthiazol-2-yl)-2,5-diphenyltetrazolium bromide
OD	Optical density
PBS	Phosphate-buffered saline
TNF	Tumor necrosis factor
TSB	Tryptic soy broth
