# Supplementary material for: Cell-Free Culture Supernatant of Lactobacillus acidophilus AG01 and Bifidobacterium animalis subsp. lactis AG02 Reduces the Pathogenicity of NetB-Positive Clostridium perfringens in a Chicken Intestinal Epithelial Cell Line
Source: Microorganisms. 2024 Apr 22;12(4):839. doi: 10.3390/microorganisms12040839 (PMC11052021; doi:10.3390/microorganisms12040839)
Supplement: Supplementary file 1 [file microorganisms-12-00839-s001.zip › microorganisms-2874531-supplementary.pdf]

microorganisms

MDPI

Article

Cell-Free Culture Supernatant of *Lactobacillus acidophilus* AG01 and *Bifidobacterium animalis* subsp. *lactis* AG02 Reduces the Pathogenicity of NetB-Positive *Clostridium perfringens* in a Chicken Intestinal Epithelial Cell Line

Darshana Kadekar [1], Andreea Cornelia Udrea [1], Steffen Yde Bak [2], Niels Christensen [2], Kirsty Gibbs [3], Chong Shen [1,*] and Marion Bernardeau [3,4,*]

[1] Gut Immunology Lab, R&D, Health & Biosciences, IFF, 8220 Brabrand, Denmark; darsh311@gmail.com (D.K.); andreea.cornelia.udrea@iff.com (A.C.U.)
[2] IFF Advanced Analysis, R&D, ET, IFF, 8220 Brabrand, Denmark; steffen.yde.bak@iff.com (S.Y.B.); niels.christensen@iff.com (N.C.)
[3] Danisco Animal Nutrition, IFF, 2342 BH Oegstgeest, The Netherlands; kirsty.gibbs@iff.com
[4] Agro-Food Department, Normandy University, UNICAEN, ABTE, 14000 Caen, France
* Correspondence: chong.shen@iff.com (C.S.); marion.bernardeau@iff.com (M.B.)

Supplementary materials

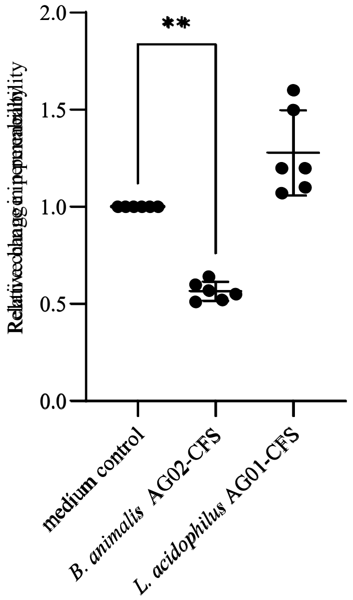

Figure S1. Effect of CFS from two probiotic strains *L. acidophilus* AG01 and *B. animalis* AG02 (30µl/ml) on CHIC-8E11 cell permeability.

Figure showing the effect of probiotic cell-free supernatants harvested from *L. acidophilus* AG01 and *B. animalis* AG02, on CHIC-8E11 permeability. Permeability is measured using the Fluorescein Isothiocyanate-dextran (FITC-D) permeability assay and is expressed as a percentage of the amount (µg) of FD4 in the basolateral compartment of cell culture inserts relative to that in the apical compartment after overnight incubation at 37°C in an atmosphere of 5% CO2. The reduction in permeability (%) was calculated relative to the medium control (without probiotic CFS pre-treatment). Experiments were performed

three times with 6 replicates. Values represent means ± SD. *, statistically significant at $P < 0.05$; **, statistically significant at $P < 0.01$; n.s., non-significant at $P < 0.05$.

Table S1. Proteins, TaqMan ID numbers and expression results for CHIC 8E11 cell line verification.

Proteins (Gene Name)	Major Cell Source	TaqMan ID No.	Expression (±)		
			CHIC 8E-11	Caco-2	IPEC-J2
	**Chicken**				
IL-10	Myeloid and lymphoid cells, granulocytes	Gg03358689_m1	–	–	–
IL-8	Macrophage, epithelial and endothelial cells, smooth muscle cells	Gg03348119_m1	+	–	–
MUC2	Goblet cells, epithelial cells	Gg03326003_m1	–	–	–
pIgR	epithelial cells	Gg03359883_m1	–	–	–
CCL5 (RANTES)	T cells, endothelial cells, epithelial cells, parenchymal cells	Gg03360168_m1	–	–	–
CXCL10 (IP-10)	leukocytes, activated neutrophils, eosinophils, monocytes, epithelial cells, endothelial cells, fibroblasts and keratinocytes	Gg03312108_m1	–	–	–
IL-1 beta	Myeloid cells, neutrophils, keratinocytes, epithelial and endothelial cells, lymphocytes, smooth muscle cells and fibroblasts	Gg03347154_g1	–	–	–
CDX1	Epithelial cell	Gg03338368_m1	+	–	–
CDX4	Epithelial cell	Gg03337886_m1	–	–	–
Villin	Enterocyte	Gg03326729_m1	–	–	–
Sox-9	Intestinal stem cells, Paneth cell	Gg03364395_m1	+	–	–
Keratin 5	Epithelial cell	Gg03345473_m1	–	–	–
Keratin 14	Epithelial cell	Gg03358651_m1	–	–	–
Vimentin	Mesenchymal cells	Gg03360310_m1	+	–	–
Desmin	Mesenchymal cells	Gg03330588_m1	+	–	–
CDH3	Epithelial cell	Gg03332134_g1	+	–	–
Lgr-5	Intestinal stem cell	Gg03327051_m1	+	–	–
Pcna	Multi-, for cell proliferation	Gg03363697_m1	+	–	–
ACTA2	Fibroblast	Gg03352404_m1	+	–	–
Olfm4	Intestinal stem cell	Gg03359583_m1	–	–	–
Znrf3	Intestinal stem cell	Gg07167606_s1	–	–	–
HopX	Intestinal stem cell	Gg03337545_m1	+	–	–
TNF-α	Myeloid, Lymphoid, epithelial cells	Gg03364359_m1	–	–	–
iNOS	T cells, macrophages, Dendritic cell and epithelial cells	Gg03347749_m1	–	–	–
IL-12B	Myeloid cells	Gg03349677_m1	–	–	–
TBP	Multi-, housekeeping	Gg03366486_m1	+	–	–
POLR2B	Multi-, housekeeping	Gg03337168_m1	+	–	–
Beta-Actin	Multi-, housekeeping	Gg03815934_s1	+	–	–
	**Human**				
MUC2	Goblet cells, epithelial cells	Hs03005103_g1	–	+	–
CLDN4	Epithelial cells	Hs00976831_s1	–	+	–
GUSB	Multi-, housekeeping	Hs99999908_m1	–	+	–
	**Pig**				
CLDN4	Epithelial cells	Ss03375006_u1	–	–	+
CD14	Myeloid cells	Ss03818718_s1	–	–	–
HPRT	Multi-, housekeeping	Ss03818718_s1	–	–	+

+, ΔCT≤ 25 cycles; –, ΔCT>25 cycles or not detectable.

Description of data: Table listing the selected proteins, associated TaqMan ID numbers and cell expression results (+/-) in CHIC-8E11 cells compared with Caco-2 and IPEC-

J2 cells, as part of the pre-experimental confirmation of IPEC-J2 cell line as a chicken cell line of intestinal epithelial origin.
